# Bioactive Immune Components of Anti-Diarrheagenic Enterotoxigenic Escherichia coli Hyperimmune Bovine Colostrum Products

**DOI:** 10.1128/CVI.00186-16

**Published:** 2017-08-04

**Authors:** Khandra T. Sears, Sharon M. Tennant, Mardi K. Reymann, Raphael Simon, Nicky Konstantopoulos, William C. Blackwelder, Eileen M. Barry, Marcela F. Pasetti

**Affiliations:** a Center for Vaccine Development, University of Maryland School of Medicine, Baltimore, Maryland, USA; b Department of Pediatrics, University of Maryland School of Medicine, Baltimore, Maryland, USA; c Department of Medicine, University of Maryland School of Medicine, Baltimore, Maryland, USA; d Immuron, Blackburn North, Victoria, Australia; University of Florida

**Keywords:** bactericidal activity, enterotoxigenic E. coli, functional antibodies, hyperimmune colostrum

## Abstract

Diarrhea is a common illness among travelers to resource-limited countries, the most prevalent attributable agent being enterotoxigenic Escherichia coli (ETEC). At this time, there are no vaccines licensed specifically for the prevention of ETEC-induced traveler's diarrhea (TD), and this has propelled investigation of alternative preventive methods. Colostrum, the first milk expressed after birthing, is rich in immunoglobulins and innate immune components for protection of newborns against infectious agents. Hyperimmune bovine colostrum (HBC) produced by immunization of cows during gestation (and containing high levels of specific antibodies) is a practical and effective prophylactic tool against gastrointestinal illnesses. A commercial HBC product, Travelan, is available for prevention of ETEC-induced diarrhea. Despite its demonstrated clinical efficacy, the underlying immune components and antimicrobial activity that contribute to protection remain undefined. We investigated innate and adaptive immune components of several commercial HBC products formulated to reduce the risk of ETEC-induced diarrhea, including Travelan and IMM-124E, a newer product that has broader gastrointestinal health benefits. The immune components measured included total and ETEC-specific IgG, total IgA, cytokines, growth factors, and lactoferrin. HBC products contained high levels of IgG specific for multiple ETEC antigens, including O-polysaccharide 78 and colonization factor antigen I (CFA/I) present in the administered vaccines. Antimicrobial activity was measured *in vitro* using novel functional assays. HBC greatly reduced ETEC motility in soft agar and exhibited bactericidal activity in the presence of complement. We have identified immune components and antimicrobial activity potentially involved in the prevention of ETEC infection by HBC *in vivo*.

## INTRODUCTION

Enterotoxigenic Escherichia coli (ETEC) is a major etiology of bacterial diarrhea in children living in resource-limited countries and the main attributable cause of diarrhea in travelers and military personnel deployed to these regions (1–3). Usually self-limiting, classic traveler's diarrhea (TD) is defined as three or more unformed bowel movements per 24 h and is often accompanied by at least one additional symptom such as stomach cramps, fever, nausea, vomiting, and blood (1). In addition to being disruptive to travel, TD causes burdensome medical expenses, loss of productivity, and important postinfectious sequelae, including irritable bowel syndrome (4, 5). The prevalence of ETEC infection and the morbidity associated with it underscore the necessity for effective preventive tools.

There is no commercially available ETEC-specific vaccine. The licensed oral cholera vaccine, Dukoral, is recommended for a secondary indication to prevent TD caused by ETEC that express heat-labile enterotoxin (LT) due to the homology between the cholera toxin B (CTB) and the ETEC LT B subunits. However, this vaccine has a modest protective efficacy (approximately 50 to 60%) against ETEC-induced TD (6, 7), and while available in Europe and Canada, Dukoral is not licensed in the United States (8). Several whole-cell and subunit ETEC vaccine candidates have been investigated in preclinical and clinical studies with various levels of success (7, 9). Progress in developing a broadly protective ETEC vaccine has been hindered by regional differences in the diversity and prevalence of ETEC serotypes, colonization factors, and other antigens (10) and by our limited understanding of the immunological effectors required to achieve protective immunity. LT along with the heat-stable toxin (ST) contribute to the extravasation of intestinal fluid and while ST is poorly immunogenic, LT neutralizing antibodies contribute to protection from illness (11, 12). Antibodies that can block host cell attachment through binding to cell surface fimbrial antigens (coli surface antigens [CSs]) and colonization factors (CF), including colonization factor antigen I (CFA/I), have been associated with reduction of symptomatic disease (13–16). However, the association between serum IgG titers and protection does not apply to all CSs (14). Further, more than 25 fimbrial antigens have been characterized, and more than one may be expressed by a particular isolate (17).

The challenges of ETEC vaccine development have spurred a search for alternative prophylactic approaches. Foremost among these have been fortified natural compounds or nutraceuticals, such as bovine colostrum, the nutrient-enriched milk produced within 24 h of birthing. Bovine colostrum contains high levels of antibodies, cytokines, growth factors, and antimicrobial peptides and passively protects newborn calves from environmental pathogens while their immune system develops (18–20). The therapeutic benefit of bovine colostrum to human health has long been recognized. In fact, bovine colostrum concentrates have been widely used as nutritional supplements and therapeutics against gastrointestinal (GI) pathogens (21, 22). Hyperimmune bovine colostrum (HBC) with high concentrations of IgG for a specific pathogen is produced by repeated immunization of pregnant cows. The use of HBC rich in microbe-specific IgG for the prevention and treatment of GI infections has the advantages of being a safe product (with standard dairy farming and manufacturing practices followed) that humans consume regularly, and unlike antibiotics, they do not disturb the gut microbiome (23).

An early clinical study conducted by Tacket et al. showed that daily consumption of an ETEC hyperimmune bovine milk concentrate shortly after each meal protected volunteers from an experimental oral challenge with 1.2 × 10^9^ CFU of ETEC virulent strain H10407 (24). In a subsequent study by Otto and colleagues, an ETEC HBC delivered prior to each meal reduced the incidence and volume of diarrheal stools in more than 90% of volunteers orally challenged with 1 × 10^9^ CFU of strain H10407 (25). These HBC products were the precursors of Travelan, an anti-ETEC HBC manufactured by Immuron Ltd. and commercially available for prophylaxis of TD in the United States, Australia, and Canada (Immuron Ltd., Blackburn North, Victoria, Australia). A similar newer product produced by Immuron, designated IMM-124E, is being tested in humans to assess gastrointestinal and systemic (i.e., liver) therapeutic benefits (ClinicalTrials.gov identifier under registration no. or identifier NCT02316717).

Apart from the demonstrated clinical efficacy of HBC in preventing enteric infections (22, 26), there is little information regarding the specific immune components and mechanisms that mediate their immune modulatory and protective activity. Hence, the goal of this study was to characterize the innate and adaptive immune components of HBC products that might contribute to their health-improving effects and disease prevention. To this end, we investigated the presence of cytokines, growth factors, lactoferrin, as well as total IgG and IgA and ETEC-specific IgG in IMM-124E powders and tablets, alongside the commercial product Travelan and nonhyperimmune bovine milk control. Most importantly, we established *in vitro* functional assays and examined the antimicrobial activity of HBC products, specifically their ability to limit ETEC motility and promote bacterial killing in the presence of complement, as possible mechanisms by which they limit ETEC-induced disease in humans.

## RESULTS

### Innate immune components in bovine colostrum products.

We initially examined the presence of nonspecific innate immune components in bovine colostrum products (BCP), including a subset of growth factors, i.e., epidermal growth factor (EGF), growth hormone (GH), insulin growth factor 1 (IGF-1), transforming growth factor beta 2 (TGF-β2), tumor necrosis factor alpha (TNF-α), interferon gamma (IFN-γ), as well as cytokines, i.e., interleukin 1 beta (IL-1β), IL-2, IL-4, and IL-6, and the antimicrobial protein lactoferrin. These components, reportedly present in unprocessed bovine colostrum and milk, were first measured in whole colostrum from healthy cows and in bovine serum to confirm that they could be adequately quantified by the commercial assays used (Table 1). All of the cytokines and growth factors tested, except IL-6 and TGF-β2, were detected in bovine serum. GH, IGF-1, IL-1β, IL-4, TNF-α, and lactoferrin were detected in unprocessed colostrum. IL-6 and TGF-β2 were not detected in colostrum or in serum, as were EGF, IFN-γ, and IL-2 (Table 1).

**TABLE 1:** Concentration of immune components in unprocessed, bovine colostrum and serum from healthy cows

Category	Immune component	Concn of immune component in^*a*^:	
		Unprocessed colostrum	Serum
Growth factors	EGF	ND	0.5 ng/ml
	GH	9.4 ng/ml	7.5 ng/ml
	IGF-1	428.1 pg/ml	404.1 pg/ml
	TGF-β2	ND	ND
Antimicrobial protein	Lactoferrin	1.6 ng/ml	0.4 ng/ml
Cytokines	IFN-γ	ND	191.2 pg/ml
	IL-1β	48.2 pg/ml	45.9 pg/ml
	IL-2	ND	284.9 pg/ml
	IL-4	87.5 pg/ml	166.9 pg/ml
	IL-6	ND	ND
	TNF-α	398.6 pg/ml	133.6 pg/ml
Ig class/subclass	Total IgG	30.6 mg/ml	15.9 mg/ml
	IgG1	7.7 mg/ml	Not tested
	IgG2	0.3 mg/ml	Not tested
	IgA	5.9 mg/ml	Not tested

a ND, not detected (below detectable levels).

We next investigated the presence of innate immune components in several HBC powders (active ingredient) and tablets (final formulation for human consumption): (i) IMM-124E 1-9, representing nine different lots of HBC powder, each derived from a group of Holstein cows immunized with either a formalin-inactivated O78 serotype ETEC vaccine strain (one-third of each group) or with a mix of multiple formalin-inactivated ETEC strains of different serotypes (two-thirds of each group); (ii) IMM-124E, a blend of the nine individual powders IMM-124E 1-9; (iii) IMM-124E tablets; and (iv) Travelan. Healthy cow milk powder and tablets (ProMilk) containing the same excipient composition but lacking the HBC active ingredient were included as controls. These bovine milk and colostrum products are described in detail in Table 2. The biomarker composition was examined comparing IMM-124E individual powders versus blended powders (IMM-124E 1-9), IMM-124E powder versus tablets, and IMM-124E tablets versus Travelan.

**TABLE 2:** Bovine milk and colostrum products used in this study

Product type	Product	Description [source]
Experimental colostrum powders	IMM-124E HBC 1-9	Nine different lots of colostrum prepared from Holstein cow cohorts hyperimmunized with ETEC single-strain or multistrain vaccines [Immuron Ltd.]
	IMM-124E	A blend of IMM-124E HBCs lots 1 to 9 [Immuron]
Control milk powders	ProMilk	A milk powder containing 85% milk proteins [Tatura Milk Industries Ltd., Australia]
Experimental colostrum tablets	IMM-124E (lots D8925 and D9718)	Composed of IMM-124E blended powder (600 mg/tablet) [Immuron]
	Travelan (lots 47058 and 17135)	Product for the reduction of risk of traveler's diarrhea and symptoms of minor GI disorders. Commercially available precursors to the HBC tablets made with IMM-124E DS [Immuron]
Control milk tablets	ProMilk (lots D8926 and D9719)	Composed of ProMilk (600 mg/tablet) [Immuron]

**FIG 1:**
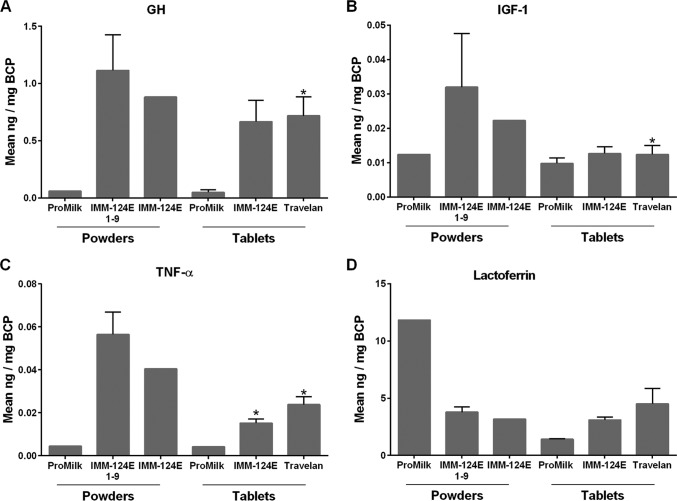
Innate immune components in bovine colostrum products (BCP). Growth hormone (GH) (A), insulin growth factor 1 (IGF-1) (B), tumor necrosis factor alpha (TNF-α) (C), and lactoferrin (D) were measured by ELISA in BCP resuspended in PBS-Tween (at a final concentration of 16 mg/ml) as described in Materials and Methods. Samples tested included ProMilk powders and tablets, IMM-124E 1-9 individual powders, IMM-124E powder and tablets, and Travelan (Table 2). Data represent the mean of duplicates when a single lot was tested (ProMilk or blended IMM-124E powder) or mean plus standard deviation (SD) (error bar) when multiple lots were available (IMM-124E HBC 1-9 [representing nine different lots of HBC powder] and two batches each of ProMilk, IMM-124E, and Travelan tablets), as listed in Table 2. Values that are statistically significantly different (*P* < 0.05) between HBC and ProMilk tablets by two-sample two-sided *t* test are indicated by an asterisk.

GH, IGF-1, and TNF-α were detected in all of the HBC products. The levels measured in the IMM-124E 1-9 individual powders were similar to those of the IMM-124E blended powder (i.e., the latter falling within 2 standard deviations [SD] of the IMM-124E 1-9 mean value), and above the ProMilk control (Fig. 1A to D). The IMM-124E powder and tablets had similar GH content, while the powder had excess concentrations of IGF-1 and TNF-α compared with the tablets (above the mean plus 2 SD of the amount measured in the tablets). All of the HBC products contained large quantities of lactoferrin. The amount of lactoferrin in IMM-124E 1-9 was similar to that of IMM-124E blended powder. An unexpected high level of lactoferrin, surpassing that of IMM-124E, was found in the ProMilk powder but not in the ProMilk tablets (Fig. 1D). Importantly, we found no statistically significant differences in the amounts of GH, IGF-1, TNF-α, and lactoferrin when comparing IMM-124E and Travelan tablets (Fig. 1). None of the remaining cytokines and growth factors tested (i.e., IFN-γ, IL-1β, IL-2, IL-4, IL-6, and TGF-β2) were detected in the HBC. EGF was found only in ProMilk and in two of the IMM-124E individual powders, at levels ranging from 0.017 to 0.063 ng/mg of BCP.

### Total and ETEC-specific antibodies in BCP.

**FIG 2:**
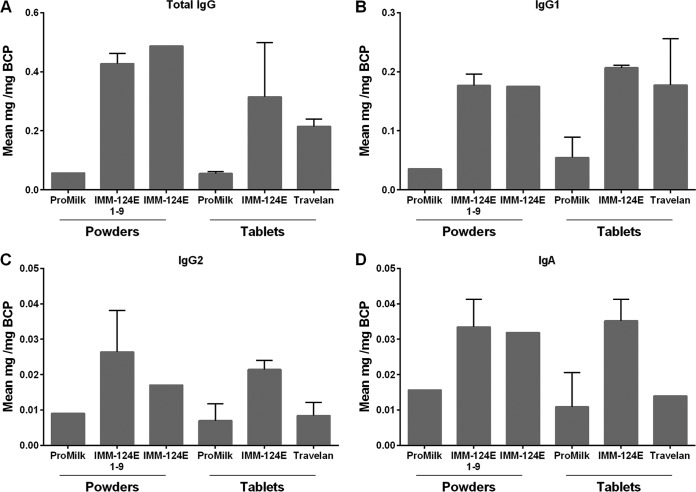
Immunoglobulin composition of BCP. Total IgG (A), IgG1 (B), IgG2 (C), and IgA (D) were measured by ELISA as described in Materials and Methods. Samples tested included ProMilk, IMM-124E, and Travelan (Table 2). Data represent Ig class or subclass content as mean of duplicates for single-lot samples or mean of multiple lots plus SD (where applicable).

We subsequently determined the total IgG, IgG1, IgG2, and IgA content in the BCP mentioned above (Fig. 2). Total IgG and IgG1 were the predominant antibody class and subclass in the HBC products (Fig. 2A and B), while IgG2 and IgA amounted to less than 10% of the total IgG in all of the samples tested (Fig. 2C and D). The antibody levels (IgG, IgG1, IgG2, and IgA) in the IMM-124E 1-9 individual powders were similar to those of the IMM-124E blended powder and above the levels found in ProMilk. The antibody levels were also similar in the IMM-124E blended powder and tablets. The levels of total IgG and IgG1 in IMM-124E tablets were not significantly different from those of Travelan. The amounts of IgG2 and IgA in IMM-124E tablets appeared higher than those of Travelan, yet the differences were not statistically significant.

**FIG 3:**
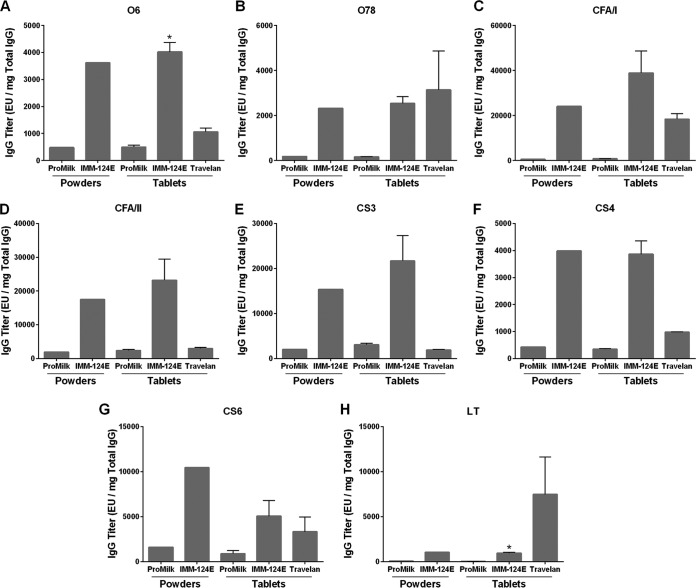
Antibody titers specific to ETEC vaccine antigens in BCP. Specific IgG antibodies to vaccine antigens O6 LPS (A), O78 COPS (B), CFA/I (C), CFA/II (D), CS3 (E), CS4 (F), CS6 (G), and E. coli LT (H) were measured by ELISA in ProMilk, IMM-124E, and Travelan. Data represent mean ELISA units (EU) (plus SD when multiple lots were tested) per milligram of total IgG. Values that are significantly different (*P* < 0.05) between HBC and ProMilk tablets by two-sided two-sample *t* test are indicated by an asterisk.

Given the similar IgG composition of the IMM-124E 1-9 individual powders and IMM-124E blended powder, ETEC-specific IgG antibodies were measured only in the blended IMM-124E HBC, as well as in the IMM-124E tablets, Travelan, and ProMilk controls (Fig. 3). IMM-124E powder and tablets had comparable levels of ETEC O6 and O78 lipopolysaccharide (LPS)-specific IgG that were above the mean level in the ProMilk controls (8- and 15-fold higher in IMM-124E tablets compared to ProMilk tablets, respectively; Fig. 3A and B). IMM-124E powder and tablets also contained CFA/I-, CFA/II-, CS3-, CS4-, and CS6-specific IgG; the levels were at least five times higher than the levels in ProMilk (Fig. 3C to G). In addition to antibodies directed to cell surface antigens, the HBC products contained LT-specific IgG at levels above those of the ProMilk control (Fig. 3H). Of note, except for O78 and LT, ETEC-specific IgG titers in IMM-124E powder and tablets surpassed those of Travelan.

**FIG 4:**
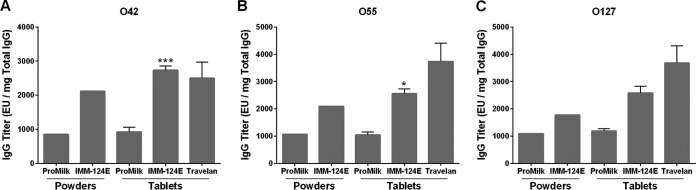
Antibody titers specific to nonvaccine ETEC antigens in BCP. Specific IgG antibodies to ETEC antigens not included in the vaccine administered to the cows were analyzed. O42 LPS (A), O55 LPS (B), and O127 LPS (C) were measured by ELISA in ProMilk, IMM-124E, and Travelan as described in the legend to Fig. 1. Data represent mean ELISA units (EU) (plus SD where applicable) of antigen-specific IgG per milligram of total IgG. Values that are statistically significantly different between HBC and ProMilk tablets by two-sided two-sample *t* test are indicated by asterisks as follows: *, *P* < 0.05; ***, *P* < 0.01.

We next examined the presence of cross-reactive antibodies to E. coli LPS serotypes not included in the vaccine (i.e., O42, O55, and O127) but known to be expressed in clinically relevant isolates. IgG recognizing these nonvaccine serotypes were detected in IMM-124E powder and tablets at levels at least twice as high as those of ProMilk tablets (Fig. 4). Cross-reactive ETEC IgG titers were also similar in IMM-124E tablets and Travelan.

We also queried the possibility of IMM-124E IgG antibodies exhibiting cross-reactive binding to LPS from other Gram-negative enteric pathogens: Shigella flexneri, Shigella sonnei, Shigella dysenteriae, and Salmonella enterica serovars Typhi, Enteritidis, and Typhimurium. IgG-recognizing Shigella and Salmonella LPS were detected in the IMM-124E powder and tablets, as well as in Travelan; the levels were at least fourfold as high as those of ProMilk (data not shown).

### Antimicrobial activity of BCP.

**FIG 5:**
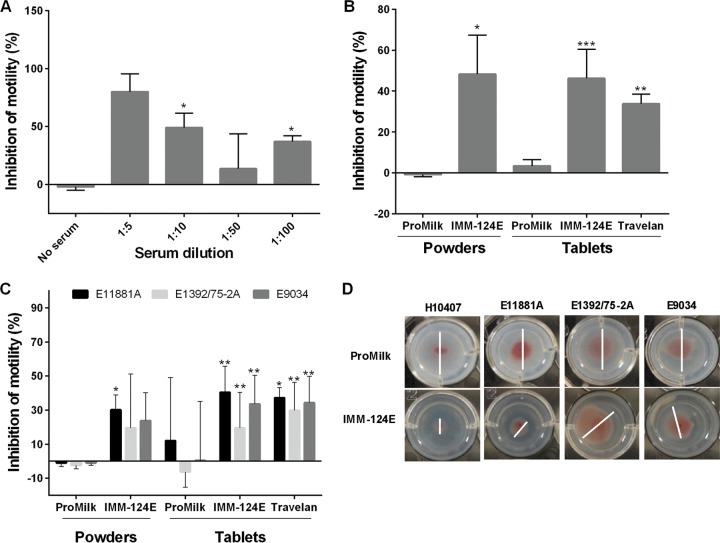
BCP inhibition of ETEC motility. Bacteria were seeded at the center of wells containing CFA agar mixed with serum or BCP. Migration was determined as the distance of growth after 4 to 6 h of incubation, and percent inhibition of motility was calculated with respect to migration in no serum or ProMilk. (A) The motility assay was optimized with immune sera (at the indicated dilutions) from volunteers challenged with strain H10407. Asterisks indicate significant differences between inhibition of motility in the presence and absence of serum. (B to D) ProMilk, IMM-124E, and Travelan were tested for their capacity to inhibit ETEC motility. (B and C) Inhibition of strain H10407 motility (B) and inhibition of strain E11881A, E1392/75/2A, and E9034 motility (C). Data shown in panels A to C represent mean percent inhibition of motility plus SD from at least three experiments; values that are significantly different between HBC and ProMilk by two-sided *t* test are indicated by asterisks as follows: *, *P* < 0.05; **, *P* < 0.01; ***, *P* < 0.001. (D) Representative images of zones of motility of ETEC strains (white bars) in agar containing ProMilk and IMM-124E.

To assess the antimicrobial activity of BCP, we developed a functional assay that allowed determination of inhibition of ETEC motility. ETEC strain H10407 was selected as the main target to measure antibody functionality because it was the strain used in multiple human challenge studies to assess vaccine and therapeutic efficacy (27). A pool of sera from volunteers challenged with strain H10407 was used as the positive (immune) control, and (nonimmune) sera from healthy individuals were used as the negative control. The motility of strain H10407 in soft agar was significantly reduced in the presence of immune sera (Fig. 5A). Further, the reduction of motility was proportional to the amount of immune sera added to the agar (Fig. 5A). Likewise, we assessed the capacity of the HBC supernatants (diluted 1:2 and embedded in agar) to inhibit ETEC motility. IMM-124E powder and tablets, as well as Travelan, significantly inhibited H10407 motility compared to ProMilk (Fig. 5B). Similar inhibition of motility was observed when testing the IMM-124E 1-9 individual powders (data not shown). The IMM-124E tablets also significantly reduced the motility of strains that express other fimbrial and O antigens present in the vaccines given to the cows, i.e., E11881A (O25:H42, CS4, CS6), E9034 (O8:H9, CS3, CS21), and E1392/75-2A (O6:H16, CFA/II) (Fig. 5C). Representative images showing inhibition of ETEC motility in the presence of IMM-124E or ProMilk are shown in Fig. 5D. The assay was performed in media that promotes expression of multiple ETEC virulence factors (28).

**FIG 6:**
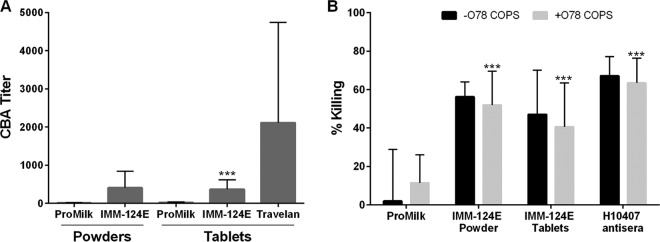
Complement-mediated colostrum bactericidal activity of BCP. ProMilk, IMM-124E, and Travelan were tested for the ability to mediate killing of strain H10407 in the presence of complement. (A) Serially diluted ProMilk, IMM-124E, and Travelan supernatants were incubated with strain H10407 in the presence of baby rabbit complement for 1 h at 37°C, and the remaining viable organisms were quantified. Endpoint titers were determined as the dilution at which 50% of bacteria were killed using Reed-Muench regression. Data represent mean bactericidal titer plus SD from three experiments. Values that are significantly different (*P* < 0.001) between HBC and ProMilk by paired *t* test or Fisher's combined probability test are indicated by asterisks. (B) Inhibition of bactericidal activity by ETEC O78. Increasing concentrations of O78 COPS were preincubated with BCP (diluted at 1:25) to capture O78-specific antibodies, and the complement killing assay was performed as described above. Data represent the percent bacteria killed with respect to bacteria and complement-only control from three experiments. Values that are significantly different (*P* < 0.001) in the presence of O78 COPS by Fisher's combined probability test are indicated by asterisks.

A bactericidal assay was established to examine the capacity of HBC antibodies to promote microbial killing in the presence of complement. Antibody-dependent complement-mediated colostrum bactericidal activity (CBA) titers were determined as the inverse of the BCP (reconstituted supernatant) dilution corresponding to 50% killing. The titers for the negative and positive (strain H10407 immune) controls were <10 and >7,000, respectively. CBA activity was first examined in the IMM124-E 1-9 individual powders; endpoint titers ranged between 124 and 270 and greatly surpassed the titer of ProMilk control (mean of <20). CBA titers were also determined in the IMM-124E blended powder and tablets, and the levels were found to be similar (407 and 369, respectively) and significantly higher than those of ProMilk (Fig. 6A). There was no statistically significant difference between the CBA activity in IMM-124E and Travelan. We did not detect consistent dose-dependent killing when HBC was tested against E11881A, E9034, and E1392/75-2A (data not shown). Given that antibodies specific for O-polysaccharide antigens are known to be involved in complement-mediated killing against other Gram-negative organisms, we attempted to determine their contribution in the observed ETEC killing. To this end, CBA titers of IMM-124E powder and tablets (supernatants) were measured after adsorption of O78 serotype-specific antibodies with increasing amounts of O78 capsular polysaccharide. Bacterial killing, though variable, was significantly reduced when O78-specific antibodies were sequestered, as observed for strain H10407 (O78) positive-control sera (Fig. 6B). Bactericidal activity of the IMM-124E powder and tablets was reduced but not completely abrogated by adsorption of O78-specific antibodies (Fig. 6B).

## DISCUSSION

Bovine colostrum is a safe and effective nutraceutical sought for the prevention and treatment of a variety of infectious diseases and immune disorders (26). HBC rich in pathogen-specific immunoglobulins has been used prophylactically to prevent illness associated with gastrointestinal infections, including ETEC-induced diarrhea (23, 26). Two products manufactured by Immuron Ltd. were the focus of our study: Travelan, a commercially available anti-ETEC HBC for prevention of TD, and IMM-124E, a more recent anti-ETEC HBC intended not only for prevention of TD but also as a therapeutic to improve systemic chronic inflammatory conditions such as metabolic syndrome and nonalcoholic steatohepatitis (NASH). Travelan has been shown to reduce both the incidence and severity of ETEC-induced diarrhea in up to 90% of volunteers (24, 25), and a similar prophylactic activity is expected of IMM-124E. While the efficacy of HBC is attributed mainly to the heightened concentration of pathogen-specific antibodies, the exact immune composition of these products and the basis for their clinical protection remain unknown. This study represents the first detailed characterization of immune components and *in vitro* antimicrobial properties of HBC; the analysis included a comparison of active ingredient and final formulation, as well as comparison of a new product and an established product with demonstrated clinical efficacy.

Our analysis of innate immune components revealed an abridged profile of cytokines (TNF-α) in HBC products compared to fresh unprocessed colostrum (IL-1β, IL-4, and TNF-α). The difference might reflect the intrinsic composition of these products (HBC was produced in New Zealand, whereas the fresh colostrum was harvested in the United States) and/or changes due to processing. The loss of components during manufacturing can affect the potency and consistency of the products, and it is therefore important to document and understand its occurrence. Our comparative analysis of IMM-124E active ingredient powder and tablets aimed precisely at identifying changes in composition due to formulation processing. The comparable results obtained for IMM-124E powder and tablets in every test performed confirmed the preservation of active ingredients in the final product. The HBC preparations contained lactoferrin, a potent antimicrobial protein capable of inhibiting ETEC growth and adherence to epithelial cells *in vitro* (29, 30). Interestingly, higher levels of lactoferrin were found in ProMilk than in the HBC powders and tablets, possibly reflecting the concentrated proteins contained in this product. Notwithstanding, ProMilk did not exhibit significant antimicrobial activity compared to the HBC products.

Aside from levels, the biological activity of the innate immune molecules detected in the HBC remains to be determined. Because of their complexity, the biological effects of milk-derived products have largely been explored using whole preparations, rather than teasing apart the roles of specific molecules. Fresh bovine colostrum and milk are known to promote growth of human intestinal cells (31), and a spray-dried colostrum product was shown to prevent gut dysfunction and inflammation in preterm pigs (32). We anticipate that the HBC products examined would exhibit similar intestinal health-restoring properties. Oral administration of IMM-124E was found to reduce local and systemic inflammation and promote peripheral regulatory R cells (Tregs) in clinical studies (47).

The HBC in this study was designed to prevent ETEC-induced diarrhea, hence the demonstration of pathogen-specific adaptive immunity was particularly relevant. The high levels of antibodies against the target vaccine antigens O6 and O78 polysaccharides, CFA/I, CFA/II, CS3, CS4, CS6, and LT detected in IMM-124E and Travelan reflect the purposeful hyperimmunization. These antibodies are believed to provide the basis for the clinical protection afforded by HBC in ETEC-challenged volunteers, which included reduced rate of illness and abdominal pain (24, 25). HBC antibodies also recognized ETEC O serotypes not included in the vaccines, including O44, O55, and O127, as well as Shigella and Salmonella O polysaccharides, likely due to the presence of shared epitopes. Furthermore, LT has homology with CT, and antibodies directed to either toxin are known to be cross-reactive. It is therefore plausible that the HBC studied might have a beneficial effect in preventing infections by other (non-ETEC) enteric pathogens.

An important contribution of this study is the demonstration of functional antimicrobial activity of HBC products, attributed mainly to ETEC-specific antibodies. We are the first to describe assays to determine inhibition of ETEC motility and complement-dependent bacterial killing and to demonstrate the presence of functional antibodies in human ETEC-immune sera and anti-ETEC HBC. Antibody activity, as opposed to antigen-binding levels, more faithfully reflects the capacity of antibodies to block organisms and promote their clearance *in vivo*. Functional assays are also valuable for confirming the biological competency of the HBC active ingredients and may be useful quality control tools for the manufacturing process. The antimicrobial activity for different lots of the same HBC product (IMM-124E and Travelan) was noticeably different, which emphasizes the importance of individual lot analysis and confirms the suitability of the assay to discriminate biological activity of different preparations.

We investigated antibody-mediated inhibition of motility because, along with adherence, it is a key step of ETEC pathogenesis (33). Antibodies specific for flagellin and fimbriae are likely responsible for inhibiting ETEC motility, although antibodies to CFA/I may also contribute to abrogating motility (34). Antibodies to LPS, which reportedly inhibited Vibrio cholerae motility (35) may also play a role. Inhibition of ETEC motility by IMM-124E tablets and Travelan varied depending on the strain, with higher percent inhibition detected against strain H10407. Such enhanced activity likely reflects the vaccine composition (one-third of the herd received exclusively formalin-inactivated H10407, while the remainder received a mix of other strains) or antigenic dominance, which ultimately resulted in an abundance of H10407-specific antibodies.

IMM-124E and Travelan also promoted killing of strain H10407 in the presence of complement. The O polysaccharide is a known target for bactericidal antibodies (36, 37). We confirmed that this is also true for ETEC, as bacterial killing activity of immune serum and HBC significantly declined, commensurate with serotype-specific antibody adsorption of O78 polysaccharide antigen prior to incubation with bacteria and complement. The fact that killing could not be completely abrogated, even for the O78-specific human sera and using larger amounts of O78, suggests that additional antigens might also be targets for antibody-mediated killing. The analysis of specificity of the functional antibody activity deserves further study. The observed lack of colostrum killing activity of strains other than H10407 may be due to the relative amount of antibodies or differences in strain biology, in particular the accessibility of antigens, which could also affect reduction of motility. LPS chain length is known to affect E. coli sensitivity to complement-mediated killing (40, 41). Similarly, serogroup-specific differences have been documented in antibody- and complement-mediated killing of Neisseria meningitidis (42).

Serum bactericidal antibody levels have been associated with clinical protection against diarrhea and illness caused by other enteric bacteria such as Shigella and V. cholerae (36, 38, 39). It would be important to determine whether the inhibition of ETEC motility and bactericidal activity observed is associated with disease prevention *in vivo*. Another important question is whether these antimicrobial functions actually represent the mechanisms of protection. Antibody binding to surface antigens may be sufficient to interfere with motility and attachment to host receptors on the gut epithelium. The extent to which bovine antibodies effectively activate human complement in the intestinal lumen is unknown.

In summary, HBC used for prevention of ETEC-induced diarrhea contains cytokines, growth factors, and lactoferrin that provide innate immune defenses and promote intestinal tissue growth and repair. In addition, HBC contains high levels of ETEC-specific antibodies, primarily IgG, including antibodies to key virulence factors. These antibodies have the capacity to inhibit ETEC motility and promote complement-mediated lysis *in vitro*. These findings provide insights into HBC innate immune components and antibody-mediated antimicrobial activities that help prevent TD. The functional antibody assays developed will be useful in monitoring immunity following infection and vaccination. These assays can also be tools to ensure quality of HBC and antibody-based immunotherapies. Because of their safety profile, demonstrated biological activity and clinical efficacy, anti-ETEC HBC represents a unique, natural, and efficacious product to prevent TD.

## MATERIALS AND METHODS

### Cow immunization and colostrum samples.

Immuron maintains a herd of approximately 7,000 Holstein-Friesian cows. Pregnant cows were immunized with either a single strain of E. coli or a multistrain E. coli vaccine before calving; approximately one-third of the cows received the single-strain vaccine, while the remaining two-thirds of the cows received the multistrain vaccine. The single-strain vaccine consisted of formalin-inactivated extracts of an E. coli serotype O78 strain (H10407), and the multistrain vaccine consisted of formalin-inactivated extracts of multiple E. coli strains (and serotypes), including B2C (O6), C55 3/3c3 (O8), PE 595 (O15), E11881A (O25), C1064-77 (O27), PE 672 (O63), E20738/0 (O114), PE 724 (O115), EI 37-2 (O128), B7A (O148), E8772/0 (O153), and PE 768 (O159). The single-strain vaccine was administered intramuscularly in three 1-ml doses, each containing 10^8^ vaccine particles. The multistrain vaccine was delivered subcutaneously in three 1-ml doses, each containing 10^8^ vaccine particles per strain. The vaccines were given 9 to 12 weeks before calving and then at 6 and 4 weeks before calving. The immunization protocol was approved by the Office of the Chief Veterinary Officer, Victoria, Australia, and conformed to the Australian Code of Practice for the Care and Use of Animals for Scientific Purposes.

Colostrum samples from multiple milkings were collected within 24 h of calving from animals that were immunized with either the single-strain vaccine or the multistrain vaccine. All colostrum samples were pooled and pasteurized with a single heat treatment of at least 72°C for a minimum of 15 s. Fat and cream were removed prior to spray drying to produce the powders. The samples investigated included (listed in Table 2): (i) commercial bovine sera from New Zealand cows (Thermo Fisher Scientific, Asheville, NC, USA); (ii) unprocessed bovine colostrum obtained from Holstein cows (Department of Animal Science, Cornell University) less than 24 h postcalving; (iii) IMM-124E 1-9 individual powders, each representing mixed colostrum from different groups of vaccinated cows; (iv) IMM-124E, a blend of all nine individual powders; (v) IMM-124E tablets (lots D8925 and D9718), (vi) Travelan tablets (lots 47058 and 17135); (vii) ProMilk 85 100-1, a commercial powdered milk concentrate (Tatura Milk Industries Ltd., Victoria, Australia); and (viii) ProMilk tablets (lots D8926 and D9719).

### Colostrum preparation.

For enzyme-linked immunosorbent assays (ELISAs), bovine colostrum and milk powders were dissolved in phosphate-buffered saline (PBS) with Tween 20 (PBS-Tween) (pH 7.4) (to a final concentration of 16 mg/ml), with shaking at room temperature for 2 h, and supernatants were collected after centrifugation at 4,000 × *g* for 10 min. For functional assays, BCP were dissolved in PBS (pH 7.4) (to a final concentration of 64 mg/ml), and supernatants were obtained as described above. Samples were stored at 4°C and were used within 2 weeks of preparation.

### Bacterial antigens and strains.

CFA/I, CFA/II, CS4, and O78 core O polysaccharide (COPS) were purified from recombinant E. coli strains at the Center for Vaccine Development (CVD) (43, 44). S. dysenteriae LPS was also produced at the CVD from strain CVD 1251 (45). The remaining antigens were obtained from commercial vendors: CS3 and CS6 (BEI Resources, Manassas, VA, USA), LT (lot 1735; Berna Biotech, Switzerland), O6, O42, O55, and O127 LPS (Sigma-Aldrich, St. Louis, MO, USA) Salmonella Enteritidis and Typhimurium LPS (Sigma-Aldrich), *S*. Typhi LPS (Becton Dickinson-Difco, Franklin Lakes, NJ, USA), S. flexneri 2a strain 2457T LPS (WRAIR), and S. sonnei strain 53G LPS (WRAIR). ETEC strains H10407 (O78:H11, LT^+^ ST^+^, CFA/I), E1392/75-2A (O6:H16, LT^−^ ST^−^, CFA/II), and E9034 (O8:H9, LT^+^, CS3, CS21) and E11881A (O25:H42, LT^+^ ST^+^, CS4, CS6) were obtained from CVD collections. Strains were maintained on tryptic soy agar (TSA) and grown on CFA medium (containing 1% [wt/vol] Casamino Acids, 0.15% [wt/vol] yeast extract, 0.005% [wt/vol] MgSO_4_, 0.0005% [wt/vol] MnCl_2_) at indicated times for functional antibody assays.

### Measurement of antibodies.

Total IgG, IgG1, IgG2, and IgA titers were measured using ELISA kits (Bethyl Laboratories, Montgomery, TX, USA). Single dilutions of each sample were tested in duplicate, and endpoint titers were calculated by extrapolation on standard curves of known immunoglobulin concentrations. Colostrum IgG antibodies specific for the following ETEC, Salmonella, and Shigella antigens were measured by ELISA using the indicated coating concentrations: CFA/I (7 μg/ml), CFA/II (5 μg/ml), CS3 (1 μg/ml), CS4 (1 μg/ml), CS6 (1 μg/ml), LT (1 μg/ml), O6 LPS (5 μg/ml), O42 LPS (5 μg/ml), O55 LPS (5 μg/ml), O78 COPS (2.5 μg/ml), O127 (5 μg/ml), Salmonella Enteritidis LPS (5 μg/ml), *S*. Typhimurium LPS (2 μg/ml), *S*. Typhi LPS (10 μg/ml), Shigella dysenteriae LPS (5 μg/ml), S. flexneri 2a strain 2457T LPS (5 μg/ml), and S. sonnei strain 53G LPS (5 μg/ml). A pool of sera from volunteers challenged with the ETEC strain H10407 was used as the positive (immune) control, and (nonimmune) sera from healthy individuals were used as the negative control (prepared from archived specimens collected during CVD study E. coli CVD-13002, unpublished data) for CFA/I and O78. For the other antigens, positive (immune) and negative (nonimmune) serum pools were prepared from archived specimens collected during various CVD clinical and preclinical studies.

CF/CS diluted in PBS (pH 7.4) was added to Immulon 2HB 96-well plates (Thermo Fisher Scientific), while LPS and COPS diluted in carbonate-bicarbonate buffer (pH 9.6) were added to medium binding microplates (Grenier Bio-One, Monroe, NC, USA). The wells on the plates were coated for 3 h, washed, and blocked overnight at 4°C (CF/CS antigens) or for 2 h at 37°C (LPS antigens). Wells used to test bovine samples were blocked with 5% (wt/vol) casein-PBS buffer, and wells used for human controls were blocked with 10% (wt/vol) nonfat dry skim milk (NFDM) (Nestle, Salon, OH, USA). HBC suspensions were diluted in PBS-Tween (0.1% [vol/vol]) with casein (0.5% [wt/vol]) (Sigma-Aldrich), and human serum control pools were diluted in PBS-Tween (0.05% [vol/vol] Tween in PBS) containing 10% (wt/vol) NFDM; samples were added to the plates and incubated for 1 h at 37°C. Specific antibodies were detected using horseradish peroxidase (HRP)-labeled goat anti-bovine IgG antibody diluted at 1:500 (Kirkegaard & Perry Laboratories, Inc. [KPL], Gaithersburg, MD, USA) in PBS-Tween with 0.5% (wt/vol) casein or HRP-labeled goat anti-human IgG antibody diluted at 1:10,000 (Jackson ImmunoResearch, West Grove, PA, USA) in PBS-Tween with 10% (wt/vol) NFDM followed by 3,3′,5,5′-tetramethylbenzidine (TMB) microwell peroxidase substrate (KPL). Absorbance values at 450 nm were measured on a microplate spectrophotometer (Multiskan FC Ascent; Life Technologies Corp., Carlsbad, CA, USA). Endpoint titers (ELISA units [EU] per milliliter for antigen-specific antibodies) were calculated as the inverse of the serum dilutions that produced an absorbance value at 450 nm (*A*_450_) of 0.2 above the blank. Antibody levels are reported as ELISA units per mg of total IgG.

### Measurement of cytokines.

Epidermal growth factor (EGF) (CUSABIO Biotech, Hubei, People's Republic of China [PRC]), lactoferrin (Bethyl Laboratories), growth hormone (GH), interferon gamma (IFN-γ), interleukin (IL) 1 beta (IL-1β), IL-2, IL-4, IL-6, insulin-like growth factor (IGF-1), transforming growth factor beta 2 (TGF-β2), and tumor necrosis factor alpha (TNF-α) (Cloud-Clone Corp., Hubei, PRC) were measured using ELISA kits following the manufacturers' instructions. *A*_450_ values were measured on a microplate spectrophotometer as described above with the exception of IFN-γ and TGF-β2 determinations, for which luminescence signals were read on a luminometer (Tecan, Switzerland) at 1 s per well. Samples were tested undiluted, in duplicate, and endpoint titers were calculated by the extrapolation of *A*_450_ values on standard curves of known concentrations of the respective analytes. Results are reported in mass (e.g., in nanograms) per milligram of reconstituted colostrum powder.

### Motility assay.

Colostrum powder supernatants were mixed 1:5 with CFA medium in a 24-well plate, eight replicates per sample. An equal volume of CFA medium with 0.6% (wt/vol) agar and 1% (wt/vol) 2,3,5-triphenyltetrazolium chloride (TTC) (Sigma-Aldrich) was added to each well to yield a final colostrum concentration of 6.4 mg/ml in 0.3% (wt/vol) agar. To prepare the assay strains, overnight cultures were grown in tryptic soy broth (TSB), then diluted 1:100 in CFA medium, and incubated at 37°C with shaking at 115 rpm until an optical density at 600 nm (OD_600_) of 0.7 to 1.0 was attained. A 1-μl drop (7 × 10^8^ to 9 × 10^8^ CFU/ml) of culture was delivered just under the surface of the agar and in the center of each well containing the colostrum-agar mix; plates were incubated in a humidifying chamber at 37°C for 4 to 6 h. Images of each plate were obtained on a Chemi Doc MP imaging system (Bio-Rad, Hercules, CA, USA). Only wells with circular or near-circular areas of growth were included for analysis. The diameter of bacterial growth in each well was determined using ImageJ software (NIH). The distance for zones of motility of hyperimmune colostrum products were calculated and expressed as a percentage of motility with respect to motility in ProMilk. Percent inhibition of motility was calculated as the difference in growth between the mean of the negative control (ProMilk) and the HBC, divided by the mean growth in the negative control multiplied by 100. The mean of a minimum of four replicates was reported as the percent inhibition of motility. Experiments were repeated at least three times with fresh milk or colostrum suspensions.

### Complement-mediated colostrum bactericidal activity assay.

The complement-mediated colostrum bactericidal activity (CBA) assay was adapted from the basic serum bactericidal assay format described by Boyd et al. (36). Briefly, colostrum powder suspensions were diluted in normal saline (0.85% sodium chloride) at 1:10 for nonhyperimmune samples and 1:25 for hyperimmune samples, 100 μl was added to each well in row A of a 96-well plate and then serially diluted twofold in saline (for a total of six dilutions). The same pools of human sera used for ELISAs were used as positive and negative controls diluted at 1:10 in saline. Samples were assayed in duplicate. Overnight cultures of E. coli H10407, E11881A, E1392/75-2A, and E9034 grown in TSB were diluted 1:1,000 in CFA medium and incubated at 37°C with shaking at 115 rpm until an OD_600_ of 0.1 to 0.2 was attained; bacteria were serially diluted in PBS for viable counts on tryptic soy agar. Baby rabbit complement (BRC) (Pel-Freez Biologicals, Rogers, AR, USA) was diluted in normal saline (5 parts BRC to 3 parts saline), and 40 μl of the complement mix was added to each well followed by 10 μl of a 10^−4^ dilution of the bacterial suspension in PBS. Each well had a final concentration of 15% BRC and approximately 40 CFU of E. coli. The wells were mixed briefly by pipetting, and the plates were incubated at 37°C with shaking at 115 rpm for 1 h. For viable counts, two 10-μl drops per well were plated on TSA plates and spread evenly by rocking; the plates were incubated at room temperature overnight. Colonies were counted, and the percent killing was calculated as (1 − mean colony count per dilution)/(mean colony count of complement-only control). Titers were reported as the dilution of colostrum at which 50% of bacteria were killed, determined using the Reed-Muench regression (46). Experiments were repeated at least three times with ProMilk or colostrum suspensions.

The contribution of O78 O polysaccharide to bactericidal activity was assessed by preincubating colostrum suspensions diluted 1:25 with 12.5 μg/ml, 25 μg/ml, and 50 μg/ml O78 COPS for 30 min at 37°C with shaking at 115 rpm. Strain H10407 and BRC were then added, and the reaction was completed as described above for s CBA. Viable counts were recorded and the percent bactericidal activity calculated as (1 − mean colony count per dilution)/(mean colony count of complement-only control).

### Statistical analysis.

The median of replicate measurements within an experiment was taken as the biomarker value for the sample. The mean was calculated for values obtained in multiple experiments. The mean levels ± 2 standard deviations (SD) for cytokines, growth factors, and antibody were determined for IMM-124E powders 1-9 (representing nine different lots of HBC powder) and considered the range of reference for these analytes in the HBC products. The mean levels ± 2 SD were compared with the levels measured in the IMM-124E blended powder and ProMilk; a value for IMM-124E blended powder or ProMilk was considered similar to the mean for IMM-124E powders 1-9 if it was within the 2 SD range. In addition, IMM-124E blended powder was considered similar to IMM-124E tablets when the value for the powder was within 2 SD of the mean for the tablets. When more than one sample was available for comparison (e.g., for two lots of IMM-124E, Travelan, and/or ProMilk tablets), they were compared using a two-sample *t* test. When more than one independent experiment was done, as for inhibition of motility and CBA titers, IMM-124E and ProMilk powders were compared by paired *t* test (one pair for each experiment) or one-sided paired *t* test within each experiment (when multiple strains were included), and Fisher's combined probability test, in which the *P* values for each experiment were combined to provide a chi-square test. IMM-124E tablets and Travelan tablets were compared to ProMilk tablets by one-sided two-sample *t* test and Fisher's combined probability test. Two-sample *t* tests allowed for unequal variances. Statistical analyses were done using Microsoft Excel and NCSS 10 software (Number Cruncher Statistical Systems, Kaysville, UT, USA). A two-sided *P* value of <0.05 was considered statistically significant. No adjustment was made for multiple comparisons.

## References

[1] Shah N, DuPont HL, Ramsey DJ 2009 Global etiology of travelers' diarrhea: systematic review from 1973 to the present. Am J Trop Med Hyg 80:609–614. 19346386

[2] Kotloff KL, Nataro JP, Blackwelder WC, Nasrin D, Farag TH, Panchalingam S, Wu Y, Sow SO, Sur D, Breiman RF, Faruque AS, Zaidi AK, Saha D, Alonso PL, Tamboura B, Sanogo D, Onwuchekwa U, Manna B, Ramamurthy T, Kanungo S, Ochieng JB, Omore R, Oundo JO, Hossain A, Das SK, Ahmed S, Qureshi S, Quadri F, Adegbola RA, Antonio M, Hossain MJ, Akinsola A, Mandomando I, Nhampossa T, Acacio S, Biswas K, O'Reilly CE, Mintz ED, Berkeley LY, Muhsen K, Sommerfelt H, Robins-Browne RM, Levine MM 2013 Burden and aetiology of diarrhoeal disease in infants and young children in developing countries (the Global Enteric Multicenter Study, GEMS): a prospective, case-control study. Lancet 382:209–222. doi:10.1016/S0140-6736(13)60844-2. 23680352

[3] Lääveri T, Antikainen J, Pakkanen SH, Kirveskari J, Kantele A 2016 Prospective study of pathogens in asymptomatic travellers and those with diarrhoea: aetiological agents revisited. Clin Microbiol Infect 22:535–541. doi:10.1016/j.cmi.2016.02.011. 26970046

[4] Wang M, Szucs TD, Steffen R 2008 Economic aspects of travelers' diarrhea. J Travel Med 15:110–118. doi:10.1111/j.1708-8305.2008.00189.x. 18346244

[5] Spiller R, Garsed K 2009 Postinfectious irritable bowel syndrome. Gastroenterology 136:1979–1988. doi:10.1053/j.gastro.2009.02.074. 19457422

[6] Scerpella EG, Sanchez JL, Mathewson JJ, Torres-Cordero JV, Sadoff JC, Svennerholm A-M, DuPont HL, Taylor DN, Ericsson CD 1995 Safety, immunogenicity, and protective efficacy of the whole-cell/recombinant B subunit (WC/rBS) oral cholera vaccine against travelers' diarrhea. J Travel Med 2:22–27. doi:10.1111/j.1708-8305.1995.tb00615.x. 9815355

[7] Venkatesan MM, Van de Verg LL 2015 Combination vaccines against diarrheal diseases. Hum Vaccin Immunother 11:1434–1448. doi:10.4161/21645515.2014.986984. 25891647 PMC4517455

[8] Centers for Disease Control and Prevention. 2016 CDC health information for international travel: the yellow book. Centers for Disease Control and Prevention, Atlanta, GA.

[9] Bourgeois AL, Wierzba TF, Walker RI 2016 Status of vaccine research and development for enterotoxigenic Escherichia coli. Vaccine 34:2880–2886. doi:10.1016/j.vaccine.2016.02.076. 26988259

[10] Fleckenstein J, Sheikh A, Qadri F 2014 Novel antigens for enterotoxigenic Escherichia coli vaccines. Expert Rev Vaccines 13:631–639. doi:10.1586/14760584.2014.905745. 24702311 PMC4199203

[11] Clemens JD, Sack DA, Harris JR, Chakraborty J, Neogy PK, Stanton B, Huda N, Khan MU, Kay BA, Khan MR, Ansaruzzaman M, Yunus M, Raghava Rao M, Svennerholm A-M, Holmgren J 1988 Cross-protection by B subunit-whole cell cholera vaccine against diarrhea associated with heat-labile toxin-producing enterotoxigenic Escherichia coli: results of a large-scale field trial. J Infect Dis 158:372–377. 3042876 10.1093/infdis/158.2.372

[12] Savarino SJ, Hall ER, Bassily S, Wierzba TF, Youssef FG, Peruski LF Jr, Abu-Elyazeed R, Rao M, Francis WM, El Mohamady H, Safwat M, Naficy AB, Svennerholm AM, Jertborn M, Lee YJ, Clemens JD, Pride Study Group. 2002 Introductory evaluation of an oral, killed whole cell enterotoxigenic Escherichia coli plus cholera toxin B subunit vaccine in Egyptian infants. Pediatr Infect Dis J 21:322–330. doi:10.1097/00006454-200204000-00012. 12075764

[13] Freedman DJ, Tacket CO, Delehanty A, Maneval DR, Nataro J, Crabb JH 1998 Milk immunoglobulin with specific activity against purified colonization factor antigens can protect against oral challenge with enterotoxigenic Escherichia coli. J Infect Dis 177:662–667. 9498445 10.1086/514227

[14] Rao MR, Wierzba TF, Savarino SJ, Abu-Elyazeed R, El-Ghoreb N, Hall ER, Naficy A, Abdel-Messih I, Frenck RW Jr, Svennerholm AM, Clemens JD 2005 Serologic correlates of protection against enterotoxigenic Escherichia coli diarrhea. J Infect Dis 191:562–570. doi:10.1086/427662. 15655780

[15] Svennerholm AM, Wenneras C, Holmgren J, McConnell MM, Rowe B 1990 Roles of different coli surface antigens of colonization factor antigen II in colonization by and protective immunogenicity of enterotoxigenic Escherichia coli in rabbits. Infect Immun 58:341–346. 1967593 10.1128/iai.58.2.341-346.1990 PMC258460

[16] Barry EM, Wang J, Wu T, Davis T, Levine MM 2006 Immunogenicity of multivalent Shigella-ETEC candidate vaccine strains in a guinea pig model. Vaccine 24:3727–3734. doi:10.1016/j.vaccine.2005.07.013. 16169130

[17] Isidean SD, Riddle MS, Savarino SJ, Porter CK 2011 A systematic review of ETEC epidemiology focusing on colonization factor and toxin expression. Vaccine 29:6167–6178. doi:10.1016/j.vaccine.2011.06.084. 21723899

[18] Wheeler TT, Hodgkinson AJ, Prosser CG, Davis SR 2007 Immune components of colostrum and milk–a historical perspective. J Mammary Gland Biol Neoplasia 12:237–247. doi:10.1007/s10911-007-9051-7. 17992474

[19] Stelwagen K, Carpenter E, Haigh B, Hodgkinson A, Wheeler TT 2009 Immune components of bovine colostrum and milk. J Anim Sci 87:3–9. doi:10.2527/jas.2008-1377. 18952725

[20] van Hooijdonk AC, Kussendrager KD, Steijns JM 2000 In vivo antimicrobial and antiviral activity of components in bovine milk and colostrum involved in non-specific defence. Br J Nutr 84(Suppl 1):S127–S134. doi:10.1017/S000711450000235X. 11242457

[21] Struff WG, Sprotte G 2007 Bovine colostrum as a biologic in clinical medicine: a review. Part I: biotechnological standards, pharmacodynamic and pharmacokinetic characteristics and principles of treatment. Int J Clin Pharmacol Ther 45:193–202. 17474538 10.5414/cpp45193

[22] Struff WG, Sprotte G 2008 Bovine colostrum as a biologic in clinical medicine: a review. Part II: clinical studies. Int J Clin Pharmacol Ther 46:211–225. doi:10.5414/CPP46211. 18538107

[23] Steele J, Sponseller J, Schmidt D, Cohen O, Tzipori S 2013 Hyperimmune bovine colostrum for treatment of GI infections: a review and update on Clostridium difficile. Hum Vaccin Immunother 9:1565–1568. doi:10.4161/hv.24078. 23435084

[24] Tacket CO, Losonsky G, Link H, Hoang Y, Guesry P, Hilpert H, Levine MM 1988 Protection by milk immunoglobulin concentrate against oral challenge with enterotoxigenic Escherichia coli. N Engl J Med 318:1240–1243. doi:10.1056/NEJM198805123181904. 3283555

[25] Otto W, Najnigier B, Stelmasiak T, Robins-Browne RM 2011 Randomized control trials using a tablet formulation of hyperimmune bovine colostrum to prevent diarrhea caused by enterotoxigenic Escherichia coli in volunteers. Scand J Gastroenterol 46:862–868. doi:10.3109/00365521.2011.574726. 21526980 PMC3154584

[26] Rathe M, Muller K, Sangild PT, Husby S 2014 Clinical applications of bovine colostrum therapy: a systematic review. Nutr Rev 72:237–254. doi:10.1111/nure.12089. 24571383

[27] Porter CK, Riddle MS, Tribble DR, Louis Bougeois A, McKenzie R, Isidean SD, Sebeny P, Savarino SJ 2011 A systematic review of experimental infections with enterotoxigenic Escherichia coli (ETEC). Vaccine 29:5869–5885. doi:10.1016/j.vaccine.2011.05.021. 21616116

[28] Ghosh AR, Sen D, Sack DA, Hoque AT 1993 Evaluation of conventional media for detection of colonization factor antigens of enterotoxigenic Escherichia coli. J Clin Microbiol 31:2163–2166. 8370745 10.1128/jcm.31.8.2163-2166.1993 PMC265715

[29] Hoek KS, Milne JM, Grieve PA, Dionysius DA, Smith R 1997 Antibacterial activity in bovine lactoferrin-derived peptides. Antimicrob Agents Chemother 41:54–59. 8980754 10.1128/aac.41.1.54 PMC163659

[30] de Oliveira IR, Bessler HC, Bao SN, Lima RDL, Giugliano LG 2007 Inhibition of enterotoxigenic Escherichia coli (ETEC) adhesion to Caco-2 cells by human milk and its immunoglobulin and non-immunoglobulin fractions. Braz J Microbiol 38:86–92. doi:10.1590/S1517-83822007000100018.

[31] Purup S, Vestergaard M, Pedersen LO, Sejrsen K 2007 Biological activity of bovine milk on proliferation of human intestinal cells. J Dairy Res 74:58–65. doi:10.1017/S0022029906002093. 16978432

[32] Sty AC, Sangild PT, Skovgaard K, Thymann T, Bjerre M, Chatterton DE, Purup S, Boye M, Heegaard PM 2016 Spray dried, pasteurised bovine colostrum protects against gut dysfunction and inflammation in preterm pigs. J Pediatr Gastroenterol Nutr 63:280–287. doi:10.1097/MPG.0000000000001056. 26756878

[33] Croxen MA, Law RJ, Scholz R, Keeney KM, Wlodarska M, Finlay BB 2013 Recent advances in understanding enteric pathogenic Escherichia coli. Clin Microbiol Rev 26:822–880. doi:10.1128/CMR.00022-13. 24092857 PMC3811233

[34] Cao L, Zhiyong S, Timothy L, SangMu J, Muhammedin D, Carol R, Laura K, Recep A, Xinghong Y 2012 Role of overexpressed CFA/I fimbriae in bacterial swimming. Phys Biol 9:036005. doi:10.1088/1478-3975/9/3/036005. 22562964 PMC3389226

[35] Wang Z, Lazinski DW, Camilli A 29 12 2016 Immunity provided by an outer membrane vesicle cholera vaccine is due to O-antigen-specific antibodies inhibiting bacterial motility. Infect Immun doi:10.1128/IAI.00626-16. PMC5203661 27795359

[36] Boyd MA, Tennant SM, Saague VA, Simon R, Muhsen K, Ramachandran G, Cross AS, Galen JE, Pasetti MF, Levine MM 2014 Serum bactericidal assays to evaluate typhoidal and nontyphoidal Salmonella vaccines. Clin Vaccine Immunol 21:712–721. doi:10.1128/CVI.00115-14. 24623629 PMC4018884

[37] Trebicka E, Jacob S, Pirzai W, Hurley BP, Cherayil BJ 2013 Role of antilipopolysaccharide antibodies in serum bactericidal activity against Salmonella enterica serovar Typhimurium in healthy adults and children in the United States. Clin Vaccine Immunol 20:1491–1498. doi:10.1128/CVI.00289-13. 23803904 PMC3807195

[38] Boutonnier A, Dassy B, Dumenil R, Guenole A, Ratsitorahina M, Migliani R, Fournier JM 2003 A simple and convenient microtiter plate assay for the detection of bactericidal antibodies to Vibrio cholerae O1 and Vibrio cholerae O139. J Microbiol Methods 55:745–753. doi:10.1016/j.mimet.2003.08.010. 14607417

[39] Glass RI, Svennerholm AM, Khan MR, Huda S, Huq MI, Holmgren J 1985 Seroepidemiological studies of El Tor cholera in Bangladesh: association of serum antibody levels with protection. J Infect Dis 151:236–242. doi:10.1093/infdis/151.2.236. 3968450

[40] Grozdanov L, Zähringer U, Blum-Oehler G, Brade L, Henne A, Knirel YA, Schombel U, Schulze J, Sonnenborn U, Gottschalk G, Hacker J, Rietschel ET, Dobrindt U 2002 A single nucleotide exchange in the wzy gene is responsible for the semirough O6 lipopolysaccharide phenotype and serum sensitivity of Escherichia coli strain Nissle 1917. J Bacteriol 184:5912–5925. doi:10.1128/JB.184.21.5912-5925.2002. 12374825 PMC135379

[41] Stawski G, Nielsen L, Ørskov F, Ørskov IDA 1990 Serum sensitivity of a diversity of Escherichia coli antigenic reference strains. APMIS 98:828–838. doi:10.1111/j.1699-0463.1990.tb05003.x. 1699558

[42] McIntosh EDG, Bröker M, Wassil J, Welsch JA, Borrow R 2015 Serum bactericidal antibody assays – the role of complement in infection and immunity. Vaccine 33:4414–4421. doi:10.1016/j.vaccine.2015.07.019. 26187262

[43] Curtis B, Grassel C, Laufer RS, Sears KT, Pasetti MF, Barry EM, Simon R 2016 Simple method for purification of enterotoxigenic Escherichia coli fimbriae. Protein Expr Purif 119:130–135. doi:10.1016/j.pep.2015.11.007. 26581778 PMC4721563

[44] Watson DC, Robbins JB, Szu SC 1992 Protection of mice against Salmonella typhimurium with an O-specific polysaccharide-protein conjugate vaccine. Infect Immun 60:4679–4686. 1383154 10.1128/iai.60.11.4679-4686.1992 PMC258218

[45] Wu T, Grassel C, Levine MM, Barry EM 2011 Live attenuated Shigella dysenteriae type 1 vaccine strains overexpressing Shiga toxin B subunit. Infect Immun 79:4912–4922. doi:10.1128/IAI.05814-11. 21969003 PMC3232646

[46] Reed LJ, Muench H 1938 A simple method of estimating fifty per cent endpoints. Am J Epidemiol 27:493–497. doi:10.1093/oxfordjournals.aje.a118408.

[47] Mizrahi M, Ya'acov AB, Adar T, Lalazar G, Shabat Y, Lichtenstein Y, Ilan Y 2010 Alleviation of insulin resistance and liver damage by oral administration of IMM-124E is mediated by increased Tregs and associated with increased serum GLP-1 and adiponectin. Results of a phase I/II clinical trial, poster 652. 61st Annu Meet Am Assoc Study Liver Dis. Hepatology 52(Suppl):632A–633A. http://immuron.com/assets/Uploads/AASLD-Poster-2010-NASH-clinical-trial-final.pdf.

